# ‘Conversion Therapy’ As Degrading Treatment

**DOI:** 10.1093/ojls/gqab024

**Published:** 2021-08-01

**Authors:** Ilias Trispiotis, Craig Purshouse

**Keywords:** ‘conversion therapy’, sexual orientation, discrimination law, degrading treatment, article 3 ECHR, equality, human rights law

## Abstract

This article responds to the widespread uncertainty in UK and international human rights law over the legality of ‘conversion therapy’, a set of practices that aim to eradicate LGBTIQ+ sexualities and gender identities. The article pursues two main arguments. First, it is argued that all forms of ‘conversion therapy’ are disrespectful of the equal moral value of LGBTIQ+ people and violate specific protected areas of liberty and equality that are inherent in the idea of human dignity. Secondly, the article develops a theoretical account of degrading treatment under article 3 of the European Convention on Human Rights that illuminates the relationship between the prohibition of degrading treatment, human dignity and antidiscrimination. It is then argued that ‘conversion therapy’, in all its different forms, spawns the specific kind of degradation that UK and international human rights law prohibit. The article ends by analysing the positive state obligations that arise in this context.

## *1*. Introduction

‘Conversion therapy’, a widely discredited practice which, according to the UN, aims to ‘cure’ LGBTIQ+ people by changing or repressing non-heteronormative sexualities and gender identities,^1^ is banned in a small number of countries around the world. At the time of writing, within the Council of Europe only Malta, Germany and Albania have introduced nationwide bans on ‘conversion therapy’, either fully^2^ or partly.^3^ The practice is not banned in the UK.^4^ Beyond Europe, Mexico introduced a general national ban on ‘conversion therapy’ in 2020, whereas medical professionals are banned from providing ‘conversion therapy’ in Brazil, Ecuador and Taiwan. In the United States, so far 20 states have introduced bans on the practice,^5^ although many exempt religious counsellors and organisations from the scope of the prohibition. A similar exemption is part of the ban on ‘conversion therapy’ in Queensland, one of the three Australian jurisdictions banning the practice at the moment.^6^

Apart from the small number of states that have legislated against ‘conversion therapy’ to date, equally surprising is the scarcity of legal research in this area.^7^ Remarkably little academic commentary has been produced on the compatibility of ‘conversion therapy’ with specific human rights, such as the prohibition of torture and other cruel, inhuman or degrading treatment (CIDT). This is a curious omission, given the evidence that ‘conversion therapy’ can cause grave harm,^8^ not only to the significant number of individuals that experience it,^9^ but also to LGBTIQ+ people more broadly.^10^ Many of those who have undergone it have reported that it has caused them distress and humiliation, with high-profile films such as *Boy Erased*^11^ and *The Miseducation of Cameron Post*^12^ illuminating these harms to a wider audience. Furthermore, there is no evidence that ‘conversion therapy’ actually works.^13^ It is for those reasons that both the European Parliament^14^ and the UN have called on states to take action against ‘conversion therapy’.^15^ But such declarations, although politically useful, do not answer crucial questions, such as whether international human rights law actually requires banning ‘conversion therapy’, and why. Answering that question has major theoretical and practical value, since any discussion of the human rights implications of ‘conversion therapy’ is central to any law reform efforts.

In this context, this article aims, first, to identify the reasons why ‘conversion therapy’ is morally wrong; and, secondly, to investigate what those reasons entail for the compatibility of the practice with the absolute prohibition of torture and CIDT under the European Convention on Human Rights (ECHR) and UK and international human rights law. To achieve those aims, the article advances two theoretical arguments. First, it is argued that ‘conversion therapy’, which is fundamentally a problem of direct discrimination on the grounds of sexual orientation and gender identity, is intrinsically wrongful because it fails to respect the equal moral personhood of LGBTIQ+ people. ‘Conversion therapy’ disrespects LGBTIQ+ persons not only because it places them at real risk of grave physical and psychological harm; or only because it denies them specific freedoms related to sexuality and gender identity; or only because it depends on, and reflects, their social subordination. ‘Conversion therapy’ disrespects LGBTIQ+ persons for all those reasons, at the same time.

Secondly, does this distinctive combination of wrongs mean that all forms of ‘conversion therapy’ amount at a minimum to degrading treatment for the purposes of human rights law? Answering this question requires engagement with the evaluative terms predicating the prohibition of torture and CIDT. That is essential to show how ‘conversion therapy’ is *relevantly* similar to examples of abuse that are recognised as more typical forms of degrading treatment. For that reason, this article analyses the meaning of ‘degrading’ treatment under article 3 ECHR and argues that the term is conditioned by the ideas of human dignity and power. An act is degrading if it expresses the unequal moral worth of the other *and* if the acting person or entity has sufficient power or status over the victim such that their actions can put them down.^16^ It is also argued that the intentions of the agent and the perception of the victim are not necessary conditions of degrading treatment, whereas the consequences of ill-treatment for individual interests do play an important role.

The article then applies this account of degrading treatment to ‘physical’ forms; forcible forms; and, finally, ‘non-physical’ and non-forcible forms of ‘conversion therapy’. It is concluded that all forms of ‘conversion therapy’ amount at a minimum to degrading treatment in human rights law. Some violent ‘physical’ forms of ‘conversion therapy’ may amount to torture instead of degrading treatment, but their position within the architecture of the prohibition of torture or CIDT depends on their intensity in the context of the case. Since all forms of ‘conversion therapy’ fall within the scope of the prohibition, the article ends with an examination of the positive state obligations in this area.

Most parts of the discussion focus on article 3 ECHR. However, the overarching argument, namely that all forms of ‘conversion therapy’ amount at a minimum to degrading treatment, is not contingent on the ECHR. The article’s arguments on the wrongness of ‘conversion therapy’, the meaning of degrading treatment in human rights law and the positive state obligations arising from the relationship between those two apply more broadly to international human rights law.

Moreover, although most of the discussion focuses on sexuality, the proposed account covers ‘conversion therapies’ targeting either sexuality or gender identity, or both. We are aware that medical or therapeutic interventions related to gender identity raise their own specific ethical and socio-legal questions, but those questions fall beyond the scope of this article. That said, it is important to clarify from the outset that therapeutic interventions that do not pathologise any sexualities or gender identities but aim to provide acceptance and support to a person do not amount to ‘conversion therapy’. Such interventions lack the element of pathologisation of certain sexualities or gender identities that all forms of ‘conversion therapy’ share, and therefore fall outside its scope.^17^

The discussion unfolds in four sections. The first section pieces together a definition of ‘conversion therapy’ based on research by the UN and medical organisations. The second section analyses the wrongness of ‘conversion therapy’. Following that, the third section offers an interpretation of degrading treatment under article 3 ECHR, and then applies that interpretation to ‘conversion therapy’. The final section analyses the consequences of the claim that all forms of ‘conversion therapy’ violate the prohibition of torture or CIDT for the positive state obligations in this context.

## *2*. What Is ‘Conversion Therapy’?

This article began with the definition of ‘conversion therapy’ by the UN Independent Expert on Sexual Orientation and Gender Identity. This definition draws on work by established medical organisations, such as the definition used in the 2017 Memorandum of Understanding signed by leading UK health bodies and the NHS. There, ‘conversion therapy’ is described as:

a therapeutic approach, or any model or individual viewpoint that demonstrates any assumption that any sexual orientation or gender identity is inherently preferable to any other, and which attempts to bring about a change of sexual orientation or gender identity or seeks to supress an individual’s expression of sexual orientation or gender identity on that basis.^18^

‘Conversion therapy’ is an ‘umbrella term’ to describe ‘a multitude of practices and methods’ to change or suppress an individual’s sexuality or gender identity.^19^ Each involve ‘attempts to pathologize and erase the identity of individuals’.^21^ A wide range of practices have been reported, including ‘corrective’ rape and sexual assault,^22^ imprisonment and kidnapping,^23^ physical abuse,^24^ electroconvulsive shock treatments,^25^ hormone treatments^26^ and ‘aversion therapy’.^27^ Call these methods *physical* ‘conversion therapy’.

Not all forms of conversion therapy involve overt violence. At the other end of the scale are ‘talking’ therapies, which involve psychotherapy, peer support or pastoral counselling.^28^ Techniques utilised there include trying to make recipients behave in conformity with gender stereotypes;^29^ encouraging them to sever ties with their families; and promoting celibacy.^30^ Call these forms *non-physical* ‘conversion therapy’. There is significant evidence that the use of psychotherapy or pastoral counselling as a practice of ‘conversion therapy’ can cause grave, lifelong harm.^31^ So, the distinction between *physical* and *non-physical* forms of ‘conversion therapy’ does not downplay the harmfulness of the latter.^32^ People who have undergone such ‘therapies’ have reported ‘loss of self-esteem, anxiety, depression, social isolation, intimacy difficulty, self-hatred, shame, sexual dysfunction, suicidal ideation, and post-traumatic stress disorder’.^33^ Arguably, some forms of ‘conversion therapy’ might be difficult to classify as they constitute both physical and emotional abuse. The distinction between physical and non-physical forms is only a heuristic for the purposes of the threshold of severity set by the prohibition of torture or CIDT in human rights law. Later sections of this article will return to this point.

## *3*. Why Is ‘Conversion Therapy’ Wrong?

At first glance, ‘conversion therapy’ is morally wrong because it endangers the lives and health of LGBTIQ+ people. The reports from international organisations and healthcare professionals that were discussed above illustrate the grave injuries that ‘conversion therapy’ can inflict on LGBTIQ+ people. This is wrong in and of itself, regardless of any comparison between people on the grounds of sexuality and gender identity.

At the same time, ‘conversion therapy’ is morally wrong for reasons that stretch beyond the grave risks it poses for the bodily and psychological health of its victims.^34^ Unlike other harmful or medically negligent therapies, ‘conversion therapy’ singles out a protected socially salient group of people, ie LGBTIQ+ people, for disadvantageous treatment. So ‘conversion therapy’ is also, fundamentally, a problem of direct discrimination on the grounds of sexual orientation and gender identity. It is, moreover, a form of intrinsically wrongful discrimination, ie independent of its effects, because alongside its proved potential for causing grave physical and psychological harm, ‘conversion therapy’ is *basically* disrespectful of the equal moral personhood of LGBTIQ+ people.^35^ All forms of ‘conversion therapy’ are *basically* disrespectful because, aside from their actual effects on the victims and aside from social conventions about what counts as disrespect, they fail to recognise that all persons are of equal moral value regardless of their sexuality and gender identity. Put differently, they fail to show what Stephen Darwall has influentially called ‘recognition respect’.^36^ ‘Conversion therapies’ fail to show recognition respect because they fail to recognise that the status of LGBTIQ+ persons as persons has to be appropriately integrated in one’s deliberations about how to act. Apart from discounting the interests of LGBTIQ+ people to physical health, ‘conversion therapy’ manifests that *deliberative* failure in two other ways: it attacks core aspects of the identity of LGBTIQ+ people by denying them crucial freedoms related to sexuality and gender identity; and it unfairly subordinates them on the grounds of sexuality. Let us look at those in more detail.

First of all, the practice of ‘conversion therapy’ is disrespectful for the equal moral personhood of LGBTIQ+ people because it places less weight on some of their key autonomy interests without any good reason for discounting them.^37^ ‘Conversion therapy’ explicitly marks out LGBTIQ+ identities as inferior to heterosexuality and, as a result of that judgment, affords less consideration to the interests of LGBTIQ+ people.^38^ Thus, even though the basis of less consideration is the sexuality or gender identity of the person, the responses constitutive of less consideration are focused on the person and their interests. All forms of ‘conversion therapy’ share one *autonomy-diminishing* goal: to restrict a host of profoundly important interests in relation to sexuality and gender identity.^39^ Two are worthy of specific mention here. The first is the individual interest to develop one’s sexual attraction into sexual activity. Some forms of ‘conversion therapy’ are designed to suppress same-sex attraction; others to suppress the option to develop same-sex sexual attraction into same-sex sexual activity.^40^ Both forms aim to suppress fundamental choices that are central to personal autonomy:^41^ that is, choices that are central to the ideal of an autonomous life shaped by people’s successive choices among valuable options of sexuality and gender identity, among which homosexuality, bisexuality, transsexuality, intersexuality, queerness etc are as valuable as heterosexuality. The second is the interest to take pride in one’s sexuality and gender identity and make it part of one’s public personality instead of staying ‘in the closet’. This is another fundamental choice, which is also central to personal autonomy, because self-repression of one’s identity inhibits full participation in valuable aspects of public culture—from music to art to politics—that are influenced and permeated by diverse sexualities and gender identities.^42^ As a result of attacking those fundamental choices, ‘conversion therapy’ also diminishes self-worth because persons measure their own sense of worth according to their ability to realise their capabilities, goals and dreams.^43^ So, ‘conversion therapy’ disrespects the equal value of LGBTIQ+ people by discounting, absent any good reason, profoundly important interests that are central to their personal autonomy. This is one of the reasons why it is wrong.

At the same time, ‘conversion therapy’ is disrespectful for the equal moral personhood of LGBTIQ+ people for reasons that extend beyond the harms it inflicts on the specific individuals who are subjected to it. ‘Conversion therapy’ depends on, and reflects, the systematic disempowerment of LGBTIQ+ people that occurs in many societies. The message of ‘conversion therapy’—a message of contempt or disdain for LGBTIQ+ identities, which *can* and *ought to* be eliminated—is demeaning for all LGBTIQ+ people; even for those that never get to experience ‘conversion therapy’ themselves.^44^ This is because it reproduces, and promotes, the social images of LGBTIQ+ people as abnormal, disgusting etc which ground their pre-existing stigma.^45^ In these ways, ‘conversion therapy’ affects not only the people who are subjected to it, but also LGBTIQ+ people in general and the attitudes of other people towards them. In fact, it is hard to divorce the absence of a legal ban on ‘conversion therapy’ in most European countries from a social context of historical stigmatisation on the basis of homosexuality.^46^ Consider the hypothetical example of a similar practice with the inverse aim, namely a ‘therapy’ whose express aim is to convert heterosexual people to homosexuals. It is unlikely that such a practice would not be illegal.^47^ But people cannot function as equals in their societies if the state does not protect everyone from abusive practices, like ‘conversion therapy’, targeting sexuality and gender identity. Consider another example, real this time. States do take action against illegitimate forms of coercive interference with important aspects of individual identity, such as religion. The European Court of Human Rights (ECtHR), for instance, has repeatedly found that exploiting a power imbalance under specific circumstances, eg in a military environment,^48^ in order to coerce someone to change their religion amounts to ‘improper proselytism’, which enjoys no protection under the ECHR.^49^ Comparisons like these illustrate that ‘conversion therapy’ relies on, and reflects, a social order in which LGBTIQ+ people have less power and are shown less respect than heterosexual people, and in which their needs are marginalised.^50^ Those wider, subordinating effects of ‘conversion therapy’ furnish another decisive objection against it.

It might be objected that the arguments above apply only where LGBTIQ+ persons are forcibly subjected to ‘conversion therapy’ and not in cases where individuals choose to undergo it. If a ‘therapy’ provider has done enough to warn others about the potential risks from ‘conversion therapy’, then anyone who nevertheless chooses to undergo it is responsible for any harm they suffer. The next section will rebut this objection: ‘conversion therapy’ is among those forms of ill-treatment that human rights law prohibits in an absolute sense.^51^ Whether an individual consented to their ‘conversion’ is therefore irrelevant. What matters is whether, in light of the harmfulness of ‘conversion therapy’, the state did enough to protect people from it.

There is an additional point though. This consent-based objection is based on an overly narrow interpretation of the moral significance of choice: what matters is the fact of a person’s choice, rather than the circumstances under which a person made that choice.^52^ However, such an interpretation is misleading because a choice has elevated moral force only when the conditions under which it is made are right.^53^ As we saw above, ‘conversion therapy’ depends on a social context of historical stigmatisation on the basis of homosexuality. The relationship of that context with the pressure on many LGBTIQ+ persons to resist their sexuality or gender identity—a pressure that heterosexual, cisgender persons do not experience—has independent moral significance. To be clear, the argument here is not that consent is irrelevant in determining whether a certain conduct amounts to prohibited ill-treatment under human rights law. Indeed, certain treatments may violate article 3 ECHR precisely because they were forced on someone.^54^ The argument is that an overly narrow interpretation of the moral significance of choice, which focuses *only* on consent and overlooks the background conditions under which a decision is made, is under-inclusive. As the next section shows, a narrow account of freedom of choice would be unable to explain key parts of the case law under article 3 ECHR, where significant emphasis is placed on the circumstances under which someone was ill-treated, such as the existence of widespread and well-known prejudice against a protected group or the vulnerability of the victim, rather than on whether an individual had a choice to avoid ill-treatment. The role of such factors can be captured only by a broader account of the moral significance of choice, according to which, in order for a decision to be legitimate, the conditions have to be right before passing to whether the person’s choice or consent is sufficient. This broader account is morally preferable, but its full defence cannot be pursued further here.

One final point to emphasise is that, so far, we have argued that ‘conversion therapy’ is wrong because it disrespects the standing of LGBTIQ+ people as equals. However, it is important to eschew one misleading connotation of that idea, namely that ‘conversion therapy’ is wrong because it is based on incorrect beliefs about the moral worth of LGBTIQ+ persons. As we saw, the view that LGBTIQ+ persons are of lesser value is fundamental to many instances of ‘conversion therapy’. But not all instances of ‘conversion therapy’ necessarily rest on such a judgment of inferior status. Consider a religious group that offers ‘conversion therapy’ to save gay men from eternal damnation. Their intervention does not necessarily rely on the assumption that LGBTIQ+ people are intrinsically less valuable than others. In fact, their intervention might be taken to suggest the exact opposite, namely that *because* LGBTIQ+ people are of equal value, they deserve to be saved through their ‘treatment’. Nevertheless, even those benevolent forms of ‘conversion therapy’ that do not rely on a direct judgment about the equal value of LGBTIQ+ people as persons fail to accord them the equality of respect that their status as persons demands. This is because their interests—in relation to health and personal autonomy—are unwarrantedly taken to matter less than the interests of others; and, more specifically, less than the interests of heterosexual people in those very matters. Therefore, the wrongness of ‘conversion therapy’ does not depend on the beliefs of the ‘therapist’, but on a theory about the normative significance of being a person, which entails that certain considerations should not be taken as a reason for certain actions.

To recap, ‘conversion therapy’ is wrong because it disrespects LGBTIQ+ persons. It disrespects them not only because it places them at real risk of grave physical and psychological harm; or only because it denies them key freedoms related to sexuality and gender identity; or only because it depends on, and reflects, their social subordination. ‘Conversion therapy’ disrespects LGBTIQ+ persons for *all* those reasons, at the same time. Both by design and in effect, ‘conversion therapy’ flouts protected areas of liberty and equality which are, as the next section will further discuss, inherent in the idea of human dignity. This partial sketch of the wrongfulness of ‘conversion therapy’ is meant to offer a set of reasons that, though incomplete, is sufficient for the overall purpose of this article, namely, to support the view that all forms of ‘conversion therapy’ fall within the scope of the absolute prohibition of torture and CIDT in human rights law.

## *4*. ‘Conversion Therapy’ and the Scope of the Prohibition of Torture or Degrading Treatment

The previous section set out the reasons why ‘conversion therapy’ is a serious violation of human dignity. This section will link the discussion with the absolute prohibition of degrading treatment in human rights law. The argument unfolds in two parts. The first part analyses the meaning of ‘degrading treatment’ under article 3 ECHR. It is argued that one of the main aims of the prohibition of ‘degrading treatment’ is to protect individuals from serious violations of human dignity, which are specified in detail. Based on this interpretation of degradation, the second part analyses the reasons why *all* forms of ‘conversion therapy’ amount at a minimum to ‘degrading treatment’ for the purposes of international human rights law.

### *A*. Degrading Treatment and Human Dignity

Does ‘conversion therapy’ have to reach a particular level of severity in order to fall within the prohibition of degrading treatment? Answering this requires engagement with the meaning of severity in this context. This section focuses on the ECHR and UK law. However, for reasons that will be discussed in section 5 of this article, the proposed interpretation of degrading treatment also applies to the UN Committee Against Torture (CAT).

First, a treatment must reach ‘a minimum level of severity’ in order to fall within the scope of article 3 ECHR.^55^ The assessment of this depends on various factors,^56^ such as the nature and context of the treatment, its duration, its physical and mental effects, and the age, sex and health of the victim.^57^ Typically, the courts determine whether the threshold has been crossed by applying the *Pretty* test.^58^ The *Pretty* test includes two evaluative elements, namely the ‘severity’ of treatment and the ‘intensity’ of suffering. To determine ‘severity’, the courts focus on the intentions and conduct of the perpetrator. The courts then shift their focus from the perpetrator to the subjective experience of the victim to assess the ‘intensity’ of suffering. ‘Severity’ and ‘intensity’ are matters of degree and, as we saw, depend on the type and context of the treatment. Thus, apart from the fact that article 3 sets a high threshold of severity, it is difficult to know much else *ex ante*.^59^ It is difficult to know *ex ante* whether a treatment can cross the requisite threshold.^60^ So, going back to where we started, it is difficult to know *ex ante* whether ‘conversion therapy’ violates article 3.

This worry is exaggerated though. Indeed, there is uncertainty over the severity threshold, especially for practices like ‘conversion therapy’ that have seldom been tested before the courts. However, as the ECtHR develops its jurisprudence on article 3, the evaluative terms of ‘severity’ and ‘intensity’ are being replaced by more determinate rules.^61^ The ECtHR elaborates the standards underlying article 3 in two ways.^62^ The first is by adding presumptions,^63^ benchmarks^64^ and principles^65^ clarifying the circumstances under which a practice can violate article 3.^66^ The second is by developing a list of practices which, due to their seriousness, always violate article 3, such as that the rape of a detainee by a state official always amounts to torture.^67^

Time and again, the judgments above are taken to suggest that underlying article 3 is a predominantly *quantitative* approach,^68^ which works roughly along these lines: degrading treatment involves pain that is severe enough to reach the threshold set by article 3, but is not as severe as inhuman treatment; inhuman treatment involves more severe pain than degrading treatment, but not as severe as torture; torture involves the most severe pain and suffering, which is why it deserves a ‘special stigma’.^69^

It is important to resist this prevailing, quantitative interpretation of the threshold set by article 3. This is because the ECtHR also places significant emphasis on the purpose of the ill-treatment and its meaning for its victims in the context in which it was inflicted. More specifically, any serious violation of human dignity may be classified as degrading treatment under article 3, even when no bodily injury and no intense physical or mental suffering is involved. Based on this principle, the ECtHR has found several forms of ill-treatment, which have not caused sustained injuries or suffering, in violation of article 3. Examples include being forced to parade naked in front of other soldiers as punishment;^70^ and several cases involving forced strip searches where their purpose was to provoke feelings of humiliation.^71^ These cases involve treatments lacking severe physical or mental effects on their victims. Nevertheless, the ECtHR has found them in violation of the substantive limb of article 3. Those decisions are guided by important *qualitative*, rather than quantitative, considerations underlying the provision. To be clear, this argument does not aim to downplay the importance of quantitative considerations in the interpretation of the threshold set by article 3. It aims to highlight that severity is not determined solely through a quantitative exercise.^72^ In fact, as the following pages discuss, it is precisely due to certain frequently overlooked qualitative considerations that all ‘conversion therapies’—even in mild, ‘talking’ forms—amount to degrading treatment.

Tracking the qualitative considerations underlying article 3 depends on an analysis of the meaning of degrading treatment under the provision. The word ‘degrading’ in article 3 is an evaluative term. It requires the courts to engage with the normative significance of degradation by making certain evaluations. These include whether degradation is conditioned by other ideas, such as human dignity or power; whether the intentions of the agent and the perception of the victim matter; and what role factual components, such as the consequences of ill-treatment for individual interests, play in establishing degradation. The first two of these questions will occupy this section. The next section will sketch an answer to the third question.

What does ‘degrading’ others mean and how does it relate to human dignity? In response to this question, Jeremy Waldron has observed that the term ‘degrading’ touches on a hierarchical idea.^73^ Someone is degraded when they are treated in a way that corresponds to a lower rank than they actually have. Here, the idea of rank is an important conduit into human dignity. Dignity has also traditionally had a hierarchical reference, which surfaces in talks about ‘the dignity of a king or the dignity of a general’.^74^ As Vlastos,^75^ Waldron,^76^ Taylor^77^ and others have argued, the idea of dignity as rank also informs our understanding of *human* dignity. The difference is that the contemporary idea of human dignity involves a pattern of levelling-up by extending high-status treatment to every human. In this way, appeals to human dignity reflect the effort to accord to every person something of the ‘first-class citizenship’ that was formerly accorded to the hereditary nobilities of the past.^78^ So this is why degrading treatment violates human dignity: it involves treating some people as if they rank lower to others, contrary to our equally high moral status.^79^

Let us examine now some examples of the serious type of degradation prohibited by article 3. Consider the case of *Bouyid*, where the ECtHR held that one slap by a police officer to the face of someone in custody constituted degrading treatment, even though the victim did not experience serious physical or mental suffering.^80^ The ECtHR stressed that whenever persons are deprived of their liberty they are in ‘a situation of vulnerability’.^81^ Vulnerability here is a ‘context-sensitive’ judgment that reflects the dependency and relative powerlessness of individuals in custody.^82^ In that context, the authorities are under a duty to protect them,^83^ and any recourse to violence which has not been strictly necessary ‘diminishes human dignity and is, in principle, an infringement of … Article 3’.^84^ Under those circumstances, even one slap to the face of a person constitutes a ‘serious attack on the individual’s dignity’.^85^ The ECtHR added two more specific reasons for that finding. First, a slap to the face ‘affects the part of the person’s body which expresses his individuality, manifests his social identity and constitutes the centre of his senses—sight, speech and hearing—which are used for communication with others’.^86^ A slap to the face is therefore a particularly acute form of disrespect for the equal moral personhood of the other. Secondly, the officers were in a superior position and had power over the applicants when they slapped them. When such a power imbalance exists, even a single slap degrades the person—it puts him down. It expresses that the victim counts for less; that he is powerless under the control of law-enforcement officers and is morally inferior to them.^87^

Thus, looking closely at *Bouyid*, an act is degrading when it satisfies two conditions. First, to degrade is to treat others in a way that expresses disrespect for their equal moral worth. Treating others as if they are objects rather than human persons or denying others the minimum requirements of personal autonomy and self-respect is incompatible with the inherent dignity of persons.^88^ Secondly, to degrade also requires that the person or entity acting has sufficient power or status to put others down.^89^ Those two conditions track the close links between degrading treatment and dignity in our moral vocabulary. It is, however, important to investigate further whether, when those two conditions are satisfied, an act can be classed as degrading under article 3 even in the absence of material effects on the victims.^90^ Let us consider some more examples.

The links between degrading treatment and human dignity also emerge in *Identoba*.^91^ In *Identoba*, the ECtHR found a violation of article 3, taken in conjunction with the prohibition of discrimination under article 14, because the state authorities failed to provide adequate protection to LGBT citizens during their peaceful march on the International Day Against Homophobia.^92^ Because of inadequate police intervention, the LGBT demonstrators were subject to homophobic aggression and verbal abuse by counter-demonstrators. LGBT flags and posters were ripped apart; a big mob surrounded the demonstrators, called them ‘faggots’ and ‘perverts’, and threatened to ‘crush’ them and ‘burn them to death’.^93^

Similarly to *Bouyid*, *Identoba* shows that the classification of a treatment as ‘degrading’ under article 3 is not contingent on its effects on the victims. Even absent any physical injury or serious mental suffering, ill-treatment can still qualify as ‘degrading’ provided that it constitutes an ‘affront to human dignity’.^94^ Still, for the purposes of ‘conversion therapy’, let us focus on a key aspect of the treatment in *Identoba*. What proved significant was that the recipients of the aggression were in a precarious position because of widespread homophobic prejudice against them.^95^ It was in this context that the homophobic and transphobic abuse that they experienced had the effect of arousing feelings of fear, anguish and insecurity that were incompatible with their dignity.^96^ In such circumstances,

the question of whether or not some of the applicants sustained physical injuries of certain gravity becomes less relevant. All of the thirteen individual applicants became the target of hate speech and aggressive behaviour … Given that they were surrounded by an angry mob that outnumbered them and was uttering death threats and randomly resorting to physical assaults, demonstrating the reality of the threats, and that *a clearly distinguishable homophobic bias played the role of an aggravating factor* … the situation was already one of intense fear and anxiety. The aim of that verbal—and sporadically physical—abuse was evidently to frighten the applicants so that they would desist from their public expression of support for the LGBT community.^97^

So, wrongful discrimination is an aggravating factor when considering whether ill-treatment reaches the threshold set by article 3.^98^ The ECtHR has reiterated this principle in *MC* *and* *AC*,^99^ and in *Aghdgomelashvili*,^100^ both of which, similarly to *Identoba*, involved ill-treatment that was motivated by homophobic and/or transphobic hatred. Even in the absence of intense physical or psychological suffering, such forms of direct discrimination can amount to degrading treatment under article 3 whenever they are severe enough to constitute an affront to human dignity.^101^ Notably, those links between discrimination and degrading treatment mirror the interpretation of the prohibition of torture or CIDT by the CAT, which has emphasised that the discriminatory use of violence is a determining factor in the classification of an act as torture or CIDT.^102^

When is wrongful discrimination severe enough to constitute an ‘affront’ to human dignity in violation of article 3? An early example comes from the decision of the European Commission of Human Rights in *East African Asians*.^103^ The case involved the reimposition of immigration control on the citizens of the UK and Colonies coming from East Africa, who were henceforth not able to enter ‘the only State of which they were citizens—the United Kingdom’.^104^ A combination of two factors led the Commission to conclude that the discrimination they suffered amounted to degrading treatment. First, differential treatment on the basis of race constitutes ‘a special form of affront to human dignity’.^105^ Secondly, the applicants were ‘publicly’ disadvantaged by discriminatory legislation. The public nature of the measures against them was an additional ‘aggravating’ factor when assessing whether discrimination constitutes degrading treatment under article 3.^106^ Similarly, in *Cyprus v Turkey*, the ECtHR held that Greek Cypriots living in northern Cyprus suffered severe discriminatory restrictions on the grounds of ethnic origin, race and religion.^107^ Once again, because of the grounds on which they were discriminated against *and* because their suffered discrimination was ‘public’ (ie induced by the state^108^), the ECtHR held that it amounted to degrading treatment. A closer look at those two factors, ie the ground of discrimination and its ‘public nature’, is crucial to understand when discrimination can be severe enough to constitute an ‘affront’ to human dignity and therefore violate article 3.

First, for the purposes of ‘conversion therapy’, is sexual orientation discrimination a ‘special’ affront to human dignity, like racial discrimination? After years of evolution, the jurisprudence of the ECtHR suggests that the answer is now yes. Sexual orientation concerns ‘a most intimate’^109^ and ‘vulnerable’^110^ aspect of life. Any differential treatment based on sexual orientation requires ‘very weighty reasons’ to be justified.^111^ In *Smith and Grady*, the ECtHR held that treatment grounded on ‘a predisposed bias on the part of a heterosexual majority against a homosexual minority’ may, in principle, fall within the scope of article 3.^112^ An example of sexual orientation discrimination that amounted to degrading treatment under article 3 comes from *X v Turkey*.^113^ In *X*, the prison authorities placed an inmate in solitary confinement because they assumed that his sexual orientation put him at risk of harm from other inmates. No risk assessment was carried out and no explanation was given as to why the applicant was deprived of even limited access to outdoor activities.^114^ The ECtHR held that placing the applicant in solitary confinement—a measure reserved for inmates, unlike the applicant, charged with violent offences^115^—without adequate justification was a degrading form of sexual orientation discrimination.^116^

Cases like *X*, *Identoba*, *MC* and *Aghdgomelashvili* bring discrimination on the grounds of sexual orientation or gender identity in line with the earlier discussed cases on racial discrimination: they confirm that, under certain circumstances, wrongful direct discrimination is a special affront to human dignity in violation of article 3.^117^ When state authorities abuse LGBTIQ+ people, or when they refuse or systematically fail to protect them from abuse that they knew or ought to have known about, that is a degrading form of direct discrimination. As such, even absent any serious material effects on the victims,^118^ it violates the substantive limb of article 3 read together with article 14 ECHR. This principle rightly reflects the well-established links between discrimination and degrading treatment in international human rights law. For instance, as the UN High Commissioner for Human Rights has argued, sexual orientation discrimination can dehumanise its victims, which is often a necessary condition for torture and ill-treatment to occur.^119^

Secondly, we saw that the ‘public nature’ of discrimination is an aggravating factor when assessing whether discrimination is severe enough to fall under article 3.^120^ This factor reflects the interrelation of control and powerlessness, which is salient in the ECtHR’s interpretation of degrading treatment.^121^ Cases like *East African Asians* and *Cyprus v Turkey*, where the government institutionalises discrimination, are paradigms of some persons being openly treated as ‘objects’ in the power of the authorities.^122^ In other cases, like *Identoba*, *MC* and *Aghdgomelashvili*, questions of abuse of power emerge again, albeit in a different fashion. When the state authorities systematically fail to prevent or investigate hatred-induced violence towards LGBTIQ+ people that they knew or ought to have known about, they undermine public confidence in the state duty to keep everyone physically and morally secure.^123^ Moral security depends on having one’s moral standing recognised as a limitation to what may legitimately be done to them, and that their welfare is treated as morally important by the state.^124^ When a protected group is already the target of prejudice, the failure of the authorities to offer them reasonable protection is a paradigmatic affront to their moral standing—it stamps them with a badge of inferiority.

Some of the most egregious forms of direct discrimination degrade their victims precisely because of the open way that they deny them profoundly important autonomy interests^125^ and self-respect.^126^ This reason might not hold in cases involving non-intentional or indirect forms of discrimination, where the psychological suffering and stigma might be somewhat less.^127^ As a result, those would be captured only by article 14 and not by article 3 ECHR. Thus, this analysis does not suggest that wrongful discrimination always amounts to degrading treatment under article 3. An interpretive judgment, similar to what these pages offer, is required to determine if an instance of discrimination spawns the type of serious degradation prohibited by the provision. Apart from the ground of discrimination, another factor affecting this interpretive judgment is its ‘public nature’; although, as we saw, ‘public’ discrimination does not require that discrimination is widely publicised.^128^ The ‘public nature’ factor is just another way of expressing a paradigm feature of degradation, namely that it rests on a significant disparity in power between two parties. It is because of that power disparity that an action degrades rather than merely insults others.

One further caveat should be mentioned before we proceed: the power or status disparity in degrading treatment does not require that ill-treatment is forced on an individual. Although this is often the case, eg when ill-treatment occurs in custody, the requirement for a power/status disparity does not extinguish the possibility for individual voluntary action. In cases like *Identoba*, *MC* and *Aghdgomelashvili*, the emphasis of the Court’s interpretation is not on whether the ill-treatment in question was forced on the applicants; rather, it was on the circumstances of widespread prejudice under which individuals were ill-treated and on the fact that, under *those* circumstances, the police either outright abused, or refused to provide reasonable protection to, the individuals in question.^129^ So, as discussed earlier, although consent is not irrelevant in determining whether conduct amounts to degrading treatment, focusing *only* on individual consent detracts from an evaluation of the background conditions in which ill-treatment was inflicted. Those background conditions have independent moral significance, which stems from the aim of the prohibition of degrading treatment to protect individuals from serious violations of human dignity.

To recap, an act is degrading if it expresses the unequal moral worth of the other *and* if the person acting occupies a position of power over the victim such that their actions can put the other down. This explains why direct discrimination on the grounds of sexual orientation and gender identity can sometimes amount to degrading treatment under article 3. Before examining if ‘conversion therapy’ fulfils those conditions of degradation, two final issues need addressing: first, whether the wrongness of a degrading act depends on the intentions of the wrongdoer; and, secondly, whether it depends on its subjective perception by the victim or others.

In response to the first question, the wrongness of degrading treatment depends on the objective meaning carried by it rather than the mental state of the wrongdoer. A slap to the face of a person has a different, ie degrading, social significance when it happens in a police station rather than outside a pub. Failing to offer reasonable protection to vulnerable people from predictable hatred-induced violence has a degrading meaning when we talk about the state authorities rather than one’s next-door neighbours. The condition that degrading treatment must express that the other is not of equal moral worth is satisfied depending on the social or conventional meaning of the conduct. Thus, the intentions of the wrongdoer are not decisive for whether an act is degrading. This objective-meaning interpretation of degrading treatment emerges clearly in the case law of the ECtHR. The ECtHR has repeatedly held that the intention to debase or humiliate is not a necessary condition of degrading treatment.^130^ A finding of degrading treatment is possible even when the intention to degrade is absent. In *Gäfgen*, the officers who threatened to torture the applicant claimed that they were trying to save a child’s life.^131^ Yet their motives made no difference to the Court’s assessment, which was that torture or degrading treatment cannot be inflicted ‘even in circumstances where the life of an individual is at risk’.^132^

As for the second question, since what determines whether an act is degrading is its meaning in a particular social context, the emphasis is not on how the victim experienced their ill-treatment. This might appear counter-intuitive because the word ‘degrading’ focuses on the impact of an act on its victim. Starting from *Ireland v UK*,^133^ the ECtHR often reiterates that a treatment is degrading if it arouses in its victim ‘feelings of fear, anguish and inferiority capable of humiliating and debasing them’.^134^ In other cases, the ECtHR stresses that degrading treatment goes beyond the inevitable element of humiliation arising from ‘legitimate punishment’^135^ or ‘mandatory military service’.^136^ These terms denote that the subjective experience of ill-treatment is central to its wrongness.

That is not the only available interpretation though. The focus of degrading treatment on its impact on the victims does not mean that the term refers to their subjective experience.^137^ It refers to what happens to the person in relation to an objective standard of dignity, ie that each person is entitled to be treated as a moral equal. In the hypothetical scenario that the applicants in *Bouyid* thought that being slapped whilst in custody was well-deserved, their treatment would still be degrading. That is why the ECtHR has held that although treatment can be degrading when it humiliates, humiliation *per se* is not a necessary condition of degrading treatment.^138^ Nor is it necessary to be humiliated in the eyes of others.^139^ A homophobic crowd might not think that it is humiliating for LGBTIQ+ people to be publicly abused while police are standing by—as happened in *Identoba*. But, insofar as the police inaction expresses the unequal moral worth of the LGBTIQ+ people in question, their inaction is degrading.

By way of contrast with the prevailing quantitative interpretations of article 3, so far we have argued that the ECtHR uses the word ‘degrading’ as an important evaluative term. This section sketched answers to two key components of the complex interpretive judgments that are necessary to flesh out degrading treatment. First, we argued that an action is degrading if it expresses the unequal moral worth of the victim and if the person acting has power over the victim such that their actions can put the other down. It is for this reason that certain instances of direct discrimination amount to degrading treatment. Secondly, we argued that neither the intentions of the wrongdoer nor the subjective perception of the victim determines whether an act is degrading.

### *B*. ‘Conversion Therapy’ within the Scope of Article 3 ECHR

Is ‘conversion therapy’ an affront to human dignity that amounts to degrading treatment under article 3? Let us start with two specific forms of ‘conversion therapy’ that violate article 3. First are extreme ‘physical’ forms of ‘conversion therapy’, such as those involving rape, electroshocks, forced examinations of genitals, injections of drugs etc.^140^ Such extreme violence can cause severe physical pain and mental suffering, and therefore those forms of ‘conversion therapy’ violate article 3.^141^ Arguably, depending on their severity, such ‘physical’ forms of ‘conversion therapy’ may constitute torture rather than degrading treatment.^142^ That said, not only the severity of ill-treatment, but also its aim, determines its position within the architecture of article 3. As the ECtHR held in *Romanov*^143^ and *Cestaro*,^144^ the use of gratuitous violence that aims to debase others deserves the stigma attached to torture. Accordingly, because of their intensity and gratuitousness, violent ‘physical’ forms of ‘conversion therapy’ amount to torture rather than degrading treatment.

Forcible ‘conversion therapy’ is a second form that violates article 3.^145^ This conclusion flows from case law on forcible medical treatments. As the ECtHR held in *Herczegfalvy*, unless the forcible treatment inflicted upon a patient were a medical necessity, it amounts to degrading treatment.^146^ The UK Court of Appeal reiterated this principle in *Wilkinson*.^147^ More specifically, according to *Herczegfalvy* and *Wilkinson*, the forcible imposition of treatment on someone can be justified only when *substantial* benefits can arise from it.^148^ Such benefits must be evidenced by ‘established principles of medicine’,^149^ and would often require the cross-examination of medical practitioners.^150^ Arguably, ‘conversion therapy’ falls woefully short of this standard. There is evidence of its lasting harmful effects on the physical and mental health of LGBTIQ+ people.^151^ No health benefits arise from ‘conversion therapy’, let alone the ‘substantial’ benefits that the law requires to justify its forcible imposition. Thus, its forcible imposition on children, adolescents or adults violates article 3. This holds *regardless of* what form forcible ‘conversion therapy’ takes—eg a violent or a mild, non-physical form—and *regardless of* the age of its victims and their capacity to consent.^152^

That leaves us with mild, non-forcible forms of ‘conversion therapy’, such as non-physical, ‘talking’ sessions which pathologise certain sexualities or gender identities and attempt to eliminate them or repress their expression.^153^ If non-physical and non-forcible forms of ‘conversion therapy’ also amount to degrading treatment, then every form of ‘conversion therapy’—from its ultra-violent to its mildest ‘talking’ varieties, and in both forcible and non-forcible forms—would fall within the scope of the absolute prohibition of torture or CIDT in human rights law.

As we saw, an act is degrading if it expresses the unequal moral worth of the other and if the wrongdoer has sufficient power over the victim. Certain cases of direct sexual orientation discrimination amount to degrading treatment for precisely those reasons. Arguably, all forms of ‘conversion therapy’—not just its ‘physical’ or forcible forms—fulfil those two conditions of degradation. Treating LGBTIQ+ people as though they are of less value is an intrinsic feature of ‘conversion therapy’. Every form of the practice manifests contempt for LGBTIQ+ identities; that contempt is then acted upon through a wilful refusal to respect the equal value of the well-being of LGBTIQ+ people. This is a dignitarian harm that occurs regardless of whether the victims of ‘conversion therapy’ get injured by the practice. This is not to suggest that the deleterious consequences of ‘conversion therapy’ for the health and well-being of its victims do not matter. On the contrary, the reasons why ‘conversion therapy’ is degrading are at least partly determined by its predictable consequences for the interests of its victims.^154^ It is worth revisiting some of those predictable consequences, which were highlighted in the third section of this article, in order to flesh out why all forms of ‘conversion therapy’ amount, at a minimum, to degrading treatment.

As we discussed earlier, ‘conversion therapy’ imposes a real risk of grave, lifelong harm for the physical and mental health of its victims. Independent of any physical or psychological harm, however, ‘conversion therapy’ is also meaning-making for its victims and for LGBTIQ+ people more broadly.^155^ The expressive harms caused by ‘conversion therapy’ arise from the fact that it contemptuously disregards the interests and welfare of LGBTIQ+ people.^156^ Even when not stated explicitly, the degrading message of ‘conversion therapy’ is intelligible to the recipients because it reflects and repeats a widely understood message about sexuality norms, viz heterosexuality is ‘normal’ and desirable, whereas other gender identities or expressions of sexuality are not. This message is intelligible to its recipients because *they* are part of the same community of shared meanings as those who try to ‘convert’ them. That is why ‘conversion therapy’ is degrading even if that was not how it was meant by the ‘therapy’ provider or how it was conceived by the individual victims.^157^

In these ways, ‘conversion therapy’ is fundamentally incompatible with the sense of self-worth that we associate with human dignity. Self-worth requires that a person is secure in their identity as an individual, including as a member of those communities with which they identify. ‘Conversion therapy’ eradicates this sense of self-worth. Its constitutive aim is to limit the options of LGBTIQ+ persons in some of the most valuable and intimate spheres of life. The freedoms ‘conversion therapy’ brazenly denies would not be denied to a heterosexual person. Therefore, ‘conversion therapy’ treats LGBTIQ+ people as if they are not of equal moral worth to others; and, more specifically, not of equal worth to heterosexual persons.

So, ‘conversion therapy’ fulfils the first criterion of degrading treatment. Recall, though, that a degrading act also requires that its perpetrator has sufficient power or status over the recipient of the treatment. ‘Conversion therapy’ fulfils that condition too. A significant power imbalance is inherent in the practice. ‘Conversion therapy’ is typically offered by members of established social institutions, such as faith groups or medical experts, who hold greater power in relation to individual victims. Due to the significant disparity of status between pastors, doctors, therapists etc and individual victims, the disrespect expressed by ‘conversion therapy’ does not just insult its victims, it degrades them too. Therefore, all forms of ‘conversion therapy’ amount to degrading treatment because all combine basic disrespect for a protected group of people, ie LGBTIQ+ persons, with a significant imbalance of power or status between the parties involved.

The expressive harms of ‘conversion therapy’ encapsulate some of the practice’s most profound yet predictable consequences for the interests of its victims. Nevertheless, it is worth clarifying that the message conveyed by ‘conversion therapy’ is not the source of why the practice is wrong. As we saw, ‘conversion therapy’ disrespects the claims to equal consideration made by the equal moral personhood of all people regardless of sexuality or gender identity. The importance of being treated as equals means that any act that spurns the normative authority of equal concern implies that some people are second-class citizens. Put otherwise, ‘conversion therapy’ is degrading *because* it discounts the interests of LGBTIQ+ people absent any good reason for doing so. Its degrading character results from the way it wrongs individuals—and a respect-based account offers a plausible explanation of that wrong.

## 5. Positive State Obligations

So far, we have argued that all forms of ‘conversion therapy’ amount at a minimum to degrading treatment under the ECHR and UK human rights law. As such, all forms of ‘conversion therapy’ are absolutely prohibited and no consequentialist reasoning provided by the state or others can justify them. Where particular forms of ‘conversion therapy’ sit on the scale of article 3, ie whether particular ‘therapies’ constitute torture rather than degrading treatment, would depend on their deliberateness, the involvement of state agents, their specific purpose and the status of the victim in the context of the case.^158^

Even though our focus has been on the ECHR, the Yogyakarta Principles and the work of the UN CAT on ‘conversion therapy’ indicate that this article’s main arguments apply under international human rights law more broadly. It is worth briefly highlighting this point, although a fuller analysis cannot be pursued here. According to the Yogyakarta Principles, states are under an obligation to prohibit all forms of ‘conversion therapy’.^159^ This obligation flows from the absolute prohibition of torture or CIDT under international human rights law. The concluding observations of the UN CAT on two recent state periodic reports confirm this. Commenting on the seventh periodic report of Ecuador, the CAT called on the state to close all private centres where such ‘therapies’ are practised and hold anyone involved to account.^160^ Similarly, in its concluding observations on the fifth periodic report of China, the CAT expressed concern about reports that private and state clinics offered ‘conversion therapy’, including ‘involuntary confinement in psychiatric facilities’.^161^ Although in 2014 a Beijing court ordered one such clinic to pay compensation to a victim, the CAT criticised China’s ‘failure to clarify whether such practices are prohibited by law, have been investigated and ended, and whether the victims have received redress’.^162^ The CAT stressed that China should ban ‘conversion therapies’, as well as all other ‘forced, involuntary or otherwise coercive or abusive treatments’ against LGBTIQ+ people.^163^ This last point is crucial because it shows that the CAT attaches little significance to individual consent to such ‘therapies’: states are under a duty to outlaw all ‘abusive treatments’ targeting LGBTIQ+ people rather than just forcible ‘conversion therapy’.^164^

Moving back to the ECHR, it is clear that public authorities must not engage in the provision of ‘conversion therapy’ because that would violate article 3. This is not the end of the matter though. Article 3 generates a range of positive state duties, two of which are particularly important here.^165^ The first is the general, or framework, state duty to set up an effective system deterring and punishing acts of ill-treatment, backed up by enforcement mechanisms for the prevention, suppression and punishment of breaches.^166^ This framework duty extends to ill-treatment administered by private actors.^167^ The second is the more specific positive state duty to take operational measures when the authorities knew or ought to have known at the time of the existence of a real and immediate risk of ill-treatment against identified individuals from the acts of a third party.^168^ While the negative duty not to engage in torture or CIDT is absolute, the positive obligations arising from the prohibition are capable of modification on grounds of proportionality. That is, they must be interpreted in ways that do not impose a disproportionate burden on the authorities,^169^ and there is also latitude as to how they can be fulfilled.^170^

One important point on the operational duties arising from article 3 is that, as the Committee on the Elimination of Discrimination against Women (CEDAW) has noted, the requirement for an immediate risk of ill-treatment, which can be traced back to *Osman*,^171^ is problematic in cases of gender-based violence or abuse.^172^ This is because that requirement prevents capturing cases where successive episodes of gender-based violence against specific individuals or groups do show that the risk of ill-treatment is real, but where the wrongdoer is not in the direct vicinity of the victim. Drawing on CEDAW’s work, in *Volodina*, the ECtHR tacitly accepted that in cases of gender-based violence the standard against which operational state duties are assessed spans a wider window of time, starting from when the risk of ill-treatment is real, albeit not imminent.^173^ For that reason, states must carefully consider the particular context of the case, including any past history of violence.^174^ As Judge Pinto de Albuquerque argued, that standard is satisfied if the authorities know or ought to know that a specific group of people is subject to repeated abuse.^175^ It is posited that for exactly those reasons, that amended standard of assessment of operational state duties under article 3 is fully applicable to recurring violent or abusive practices based on sexual orientation or gender identity, such as ‘conversion therapy’.^176^

With this amendment to operational state duties in mind, let us return to the framework state duty under article 3. Recall that the framework duty refers to the primary state obligation to take legal measures designed to ensure that individuals are not subjected to proscribed ill-treatment—including ill-treatment administered by private individuals. Let us focus on how this framework duty applies to ‘conversion therapy’. The framework duty under article 3 often translates to a state duty to mobilise the criminal law against proscribed forms of ill-treatment. We must be careful here, though, because although criminal law is typically presumed to be an effective tool of deterrence and retribution,^177^ widening the web of criminalisation in the name of human rights protection carries significant risks.^178^ Criminalisation as part of the framework duty under article 3 has emerged in a wide range of cases, including rape,^179^ sexual abuse of minors,^180^ disproportionate police violence,^181^ ill-treatment in custody^182^ and domestic violence.^183^ The reasons behind the state duty to criminalise certain forms of ill-treatment are not always entirely clear.^184^ For instance, although the examples above involve physical abuse, the ECtHR has also justified the need for criminal law protection based on the argument that degrading treatment seriously affects human dignity and psychological well-being,^185^ regardless of whether injuries of a certain degree of severity have been inflicted.^186^

So, does the framework duty under article 3 require criminal law protection against ‘conversion therapy’? For the reasons discussed earlier, all forms of ‘conversion therapy’ attain the minimum level of severity to trigger the applicability of article 3 because all amount to a serious violation of human dignity: they directly discriminate against LGBTIQ+ people by placing their physical and psychological health at real risk of grave harm; and they can arouse in their victims feelings of fear, anguish and inferiority capable of debasing them.^187^ On that account, applying *Volodina* and *Myumyun* by analogy,^188^ criminalising the provision of all forms of ‘conversion therapy’ can be justified under the framework duty of article 3.^189^ At the same time, what the framework duty under article 3 requires are legal provisions that are sufficiently tailored to the human rights offence concerned. So, other options of legal action, such as civil means of redress, are also available and in fact might be preferable for non-intentional forms of ‘conversion therapy’.^190^ At any rate, the precise mix of civil and criminal law protections that would be sufficient against ‘conversion therapy’ requires further discussion and a more detailed contextual assessment that cannot be pursued here. Even so, it is unlikely that the contracting states to the ECHR can fulfil their framework duty under article 3 without adopting specific provisions against ‘conversion therapy’ that define the scope of the practice and clarify which public authorities have a duty to act against ‘therapy’ providers. Such provisions must also set out remedies, support and reporting mechanisms for victims, and also the types of interim measures that could be taken in this context. The framework duty under article 3 requires this basic legal apparatus to be firmly in place.^191^

## 6. Conclusion

This article has argued that ‘conversion therapy’ is wrong because it disrespects, in more ways than one, the equal moral value of LGBTIQ+ persons. As a serious violation of human dignity, all forms of ‘conversion therapy’ fall qualitatively within the scope of the absolute prohibition of torture or CIDT under the ECHR and UK and international human rights law. More specifically, all forms of ‘conversion therapy’—physical and non-physical, forcible and non-forcible—amount at a minimum to degrading treatment. As a result, states are under a positive obligation to take effective measures to protect LGBTIQ+ persons from the harms of ‘conversion therapy’. The first important step in that direction is introducing a ban on all forms of this practice.

Let us conclude by adding four final points on the scope of a ban on ‘conversion therapy’. First, as evidence from the UK National LGBT survey, the UN and the WHO shows,^192^ ‘conversion therapy’ often takes place outside the public eye, sometimes in spaces provided by faith organisations. Therefore, the positive state obligation to provide effective protection from ‘conversion therapy’ cannot be fully discharged if the legal response to the practice is reduced to disciplinary measures against health professionals.^193^ Secondly, given that the state obligation to ban ‘conversion therapy’ stems from the absolute prohibition of torture or CIDT in human rights law, the ban must cover all potential providers, including religious counsellors, even when they offer ‘conversion therapy’ whilst not acting as psychologists. Thirdly, since both forcible and non-forcible forms of ‘conversion therapy’ amount, at a minimum, to degrading treatment, a ban has to cover both. However, therapeutic interventions that do not pathologise any sexualities or gender identities but aim to provide acceptance and support for a person’s exploration of their identity have to be exempted.^194^ Those interventions do not constitute ‘conversion therapy’. They are not based on the assumption that some sexualities or gender identities are inherently inferior to others, and do not aim to change or suppress them for that reason.

Finally, this analysis does not suggest that a legal ban on ‘conversion therapy’ alone can eradicate this practice or provide sufficient protection for LGBTIQ+ people. Other steps that ought to be considered include specific protections for children and vulnerable adults, including provisions in relation to parents, legal guardians and the education context;^195^ support for survivors;^196^ public communications campaigns; and outreach programmes involving religious and community groups. Even though a ban on all forms of ‘conversion therapy’ needs to be accompanied by additional measures, the focus of this article was on the reasons why human rights law requires such a ban in the first place. Banning ‘conversion therapy’ is a vital step towards the eradication of a deeply inegalitarian practice. Its practical power is as important as its great expressive and dignitary power for the victims of ‘conversion therapy’, and for LGBTIQ+ communities all over the world.

## Footnotes

1 UN Human Rights Council, ‘Practices of So-Called “Conversion Therapy”: Report of the Independent Expert on Protection Against Violence and Discrimination Based on Sexual Orientation and Gender Identity’ (1 May 2020, A/HRC/44/53) para 2. See also Independent Forensic Expert Group, ‘Statement on Conversion Therapy’ (2020) 72 Journal of Forensic and Legal Medicine 101930, 1.

2 There is a full national ban on ‘conversion therapy’ in Malta. See Affirmation of Sexual Orientation, Gender Identity and Gender Expression Act, s 3.

3 In 2020, Germany criminalised the provision of ‘conversion therapy’ to minors. The provision of ‘conversion therapy’ to adults is outlawed provided that there is coercion, deceit or misapprehension. The Albanian Order of Psychologists prohibits registered psychologists from offering ‘conversion therapy’.

4 A private member’s bill outlawing ‘conversion therapy’ was lost when the UK Parliament was prorogued in 2019. A Memorandum of Understanding signed by the NHS and leading counselling, psychotherapy and mental health bodies have sought to end the practice in the UK. See BACP and others, ‘Memorandum of Understanding on Conversion Therapy in the UK’ (version 2, October 2017) <www.bacp.co.uk/media/6526/memorandum-of-understanding-v2-reva-jul19.pdf> accessed 26 May 2021.

5 At the time of writing, these are: New Jersey, California, Oregon, Illinois, Vermont, New Mexico, Connecticut, Rhode Island, Nevada, Washington, Hawaii, Delaware, Maryland, New Hampshire, New York, Massachusetts, Colorado, Maine, Utah and Virginia.

6 The others are Victoria and the Australian Capital Territory.

7 An exception is IY Nugraha, ‘The Compatibility of Sexual Orientation Change Efforts with International Human Rights Law’ (2017) 35 Netherlands Quarterly of Human Rights 176. ‘Conversion therapy’ involving children is mentioned in the UN Committee on the Rights of the Child, ‘General Comment No 20 on the Implementation of the Rights of the Chid During Adolescence’ (CRC/C/GC/20, 6 December 2016) para 34.

8 ‘Practices of So-Called “Conversion Therapy”’ (n 1); BACP and others (n 4); A Bartlett, G Smith and M King, ‘The Response of Mental Health Professionals to Clients Seeking Help to Change or Redirect Same-Sex Sexual Orientation’ (2009) 9(11) BioMed Central Psychiatry 7.

9 According to the 2018 UK National LGBT survey, 2% of respondents report having undergone ‘conversion therapy’ and 5% report that they had been offered it. Crucially, LGBT individuals from particular cultural, religious and age backgrounds are many times more likely to being offered ‘conversion therapy’. See UK Government Equalities Office, *National LGBT Survey: Research Report* (July 2018) 33 and 83–92.

10 ‘Practices of So-Called “Conversion Therapy”’ (n 1); UN Human Rights Council, ‘Report of the Special Rapporteur on the Right of Everyone to the Enjoyment of the Highest Attainable Standard of Physical and Mental Health, Anand Grover’ (A/HRC/14/20, 27 April 2010) para 23.

11 J Edgerton, *Boy Erased* (Focus Features, 2018).

12 D Akhavan, *The Miseducation of Cameron Post* (Beachside Films and Parkville Pictures, 2018).

13 ‘Editorial’ (2016) 387 The Lancet 95; Independent Forensic Expert Group (n 1) 5.

14 European Parliament, Committee on Civil Liberties, Justice and Home Affairs, ‘Amendment 8 to the Report on the Situation of Fundamental Rights in the EU in 2016’ (A8-0025/8, 21 February 2018).

15 UN Joint Statement, ‘United Nations Entities Call on States to Act Urgently to End Violence and Discrimination Against Lesbian, Gay, Bisexual, Transgender and Intersex Adults, Adolescents and Children’ (September 2015); Annual Report of United Nations High Commissioner for Human Rights, ‘Discriminatory Laws and Practices and Acts of Violence Against Individuals Based on their Sexual Orientation and Gender Identity’ (A/HRC/19/41, 17 November 2011) para 56; ‘Report of the Special Rapporteur on the Question of Torture and Other Cruel, Inhuman or Degrading Treatment or Punishment’ (A/56/156, UN General Assembly 3 July 2001) para 24.

16 This account of degrading treatment is congenial to how Deborah Hellman defines ‘demeaning’ treatment in D Hellman, *When* *Is* *Discrimination Wrong?* (Harvard UP 2008) 34–58. However, there are differences between how this article explains the wrongness of degrading treatment (ie through an account of disrespect) and how Hellman explains the wrongness of demeaning treatment. This article’s penultimate section returns to this point.

17 That is why some bans expressly exempt such practices from the scope of ‘conversion therapy’. See eg the legislation adopted in Queensland (Public Health Act 2005, s 213F as amended by Health Legislation Amendment Act 2020, s 28) and Victoria (Change or Suppression (Conversion) Practices Prohibition Act 2021, s 5).

18 BACP and others (n 4) para 2.

19 ‘Practices of So-Called “Conversion Therapy”’ (n 1) para 17.

21 ibid para 19.

22 ibid paras 18 and 39.

23 ibid para 39.

24 ibid paras 39, 50 and 52.

25 ‘Report of the Special Rapporteur’ (n 15) para 24.

26 ‘Practices of So-Called “Conversion Therapy”’ (n 1) para 46.

27 ibid para 43.

28 KA Hicks, ‘“Reparative” Therapy: Whether Parental Attempts to Change a Child’s Sexual Orientation Can Constitute Child Abuse’ (1999) 49 Am U L Rev 506.

29 ‘Practices of So-Called “Conversion Therapy”’ (n 1) para 45.

30 ibid para 37.

31 J Turban and others, ‘Association Between Recalled Exposure to Gender Identity Conversion Efforts and Psychological Distress and Suicide Attempts Among Transgender Adults’ (2020) 77 JAMA Psychiatry 68; J Devlin and others, ‘Sexual Orientation Change Efforts Among Current or Former LDS Church Members’ (2015) 62 Journal of Counseling Psychology 95; D Halderman, ‘Therapeutic Antidotes: Helping Gay and Bisexual Men Recover from Conversion Therapies’ (2002) 5 Journal of Gay & Lesbian Psychotherapy 117.

32 ‘Practices of So-Called “Conversion Therapy”’ (n 1) para 55.

33 ibid para 56. See also J Fjelstrom, ‘Sexual Orientation Change Efforts and the Search for Authenticity’ (2013) 60 Journal of Homosexuality 801.

34 This analysis of the wrongness of ‘conversion therapy’ distinguishes objections from the proved potential of ‘conversion therapy’ to cause grave physical and psychological harm from objections to the practice from its inegalitarian nature. However, the two objections are linked. Pain and injury can be objected to independently of their consequences to the affected people’s options. But pain and injury are harmful also because they obstruct the pursuit of a person’s options and relationships.

35 On basic disrespect, see B Eidelson, *Discrimination and Disrespect* (OUP 2015) 84–90. See also J Wolff, ‘Fairness, Respect, and the Egalitarian Ethos’ (1998) 27 Philosophy & Public Affairs 97, 107–10.

36 S Darwall, ‘Two Kinds of Respect’ (1977) 88 Ethics 36, 38. For the purposes of this article, it is not necessary to offer a more detailed account of an egalitarian notion of respect. Rather, it suffices to show that ‘conversion therapy’ undermines respect (including self-respect) in specific ways.

37 H Frankfurt, *Necessity, Volition, and Love* (CUP 1999) 146–55.

38 N Kolodny, ‘Rule Over None II: Social Equality and the Justification of Democracy’ (2014) 42 Philosophy & Public Affairs 287.

39 Since ‘conversion therapy’ breaches autonomy-based duties, state intervention is legitimate. See J Raz, *The Morality of Freedom* (OUP 1986) 416–17.

40 The definitions of ‘conversion therapy’ in some of the existing laws against it cover practices that aim to convert, cancel or suppress sexual orientation or gender identity. See n 17 above.

41 J Gardner, ‘On the Ground of Her Sex(uality)’ (1998) 18 OJLS 167, 172–3. The question of whether sexuality constitutes an immutable characteristic or a fundamental choice cannot settle whether people are entitled to protection from ‘conversion therapy’. In either case, sexuality and gender identity are so central to self-definition that the harms of ‘conversion therapy’ amount to an attack on the autonomy of LGBTIQ+ people.

42 ibid 176–8.

43 D Réaume, ‘Discrimination and Dignity’ (2003) 63 La L Rev 645, 673; Wolff (n 34) 107; T Khaitan, ‘Dignity as an Expressive Norm: Neither Vacuous nor a Panacea’ (2012) 32 OJLS 1.

44 On the demeaning message of discrimination, see D Nejaime and RB Siegel, ‘Conscience Wars: Complicity-Based Conscience Claims in Religion and Politics’ (2015) 124 Yale LJ 2516, 2574–8.

45 M Nussbaum, *From Disgust to Humanity: Sexual Orientation and Constitutional Law* (OUP 2010) 2–26.

46 The force of this objection depends on an analysis of the socio-historical particularities which determine the meaning of an act. See Hellman (n 16) 34–59.

47 A historical example can be seen in the debates surrounding the Local Government Act 1988, s 28. See J Moran, ‘Childhood Sexuality and Education: The Case of Section 28’ (2001) 4 Sexualities 73.

48
*Larissis v Greece* App no 23372/94 (24 February 1998) para 51.

49 This argument does not suggest that there is a positive state obligation to ban all forms of proselytism. It only aims to show that the legitimacy of proselytism depends, to a significant extent, on an evaluation of the background conditions in which it takes place. See *Nasirov and Others v Azerbaijan* App no 58717/10 (20 February 2020) para 65; *Jehovah’s Witnesses of Moscow and Others v Russia* App no 302/02 (10 June 2010) para 122. More broadly, egalitarian considerations can justify restrictions on freedom of religion or belief; see I Trispiotis, ‘Religious Freedom and Religious Antidiscrimination’ (2019) 82 MLR 864.

50 S Moreau, *Faces of Inequality* (OUP 2020) 39–66; N Bamforth, ‘Sexuality and Citizenship in Contemporary Constitutional Argument’ (2012) 10 ICON 477; C Stychin, *Governing Sexuality: The Changing Politics of Citizenship and Law Reform* (Hart Publishing 2003) 12–13.

51 Art 3 makes no provision for exceptions and no derogation is permissible under Art 15(2) ECHR. See *Soering v United Kingdom* App no 14038/88 (7 July 1989) para 88.

52 This distinction draws on Scanlon’s distinction between narrow (‘forfeiture’) and broad (‘value of choice’) interpretations of the moral significance of choice. See TM Scanlon, *What We Owe to Each Other* (Harvard UP 2000) 256–67.

53 ibid 260. Under a narrow account of the moral significance of choice, the ‘background’ conditions are important only if they affect the voluntariness of choice.

54 Force-feeding (eg *Nevmerzhitsky v Ukraine* App no 54825/00 (5 April 2005)) and force-sterilisation (eg *VC* *v Slovakia* App no 18968/07 (8 November 2011)) are examples of that.

55
*Ireland v United Kingdom* (1978) 2 EHRR 25, para 162; *Tyrer v United Kingdom* (1978) 2 EHRR 1, para 30.

56
*Jalloh v Germany* App no 54810/00 (Grand Chamber, 11 July 2006) para 67. See also *Kafkaris v Cyprus* App no 21906/04 (Grand Chamber, 12 February 2008) para 95.

57 On the difficulty in outlining every condition able to influence the severity test under art 3, see *R (on the application of Q) v Secretary of State for the Home Department* [2003] EWCA Civ 364, para 60.

58
*Pretty v United Kingdom* App no 2346/02 (29 April 2002) para 52. See also *R (Pretty) v Director of Public Prosecutions* [2001] UKHL 61, para 90 (Lord Hope); *R (Watts) v Bedford Primary Care Trust* [2003] EWHC Admin 2401, para 103.

59 N Mavronicola, ‘What Is an “Absolute Right”? Deciphering Absoluteness in the Context of Article 3 of the European Convention on Human Rights’ (2012) 12 HRLR 723, 748–52.

60 D Feldman, *Civil Liberties and Human Rights in England and Wales* (2nd edn, OUP 2002) 242; H Fenwick, *Civil Liberties and Human Rights* (2nd edn, Cavendish 2004) 44–5.

61 J Waldron, ‘How to Do Things with Standards’ in U Baxi, C McCrudden and A Paliwala (eds), *Law’s Ethical, Global and Theoretical Contexts: Essays in Honour of William Twining* (CUP 2015) 335–53.

62 That process of elaboration applies to both ‘severity’ and ‘intensity’, although more to the former than the latter because the ‘intensity of suffering’ depends on the individual’s subjective experience of the practice.

63 See eg *Yavuz v Turkey* (2007) 45 EHRR 16, para 38.

64 See eg *Kalashnikov v Russia* (2003) 36 EHRR 34, para 97.

65 See eg *Aksoy v Turkey* App no 21987/93 (18 December 1996) para 63.

66 J Waldron, *Torture, Terror and Trade-Offs: Philosophy for the White House* (OUP 2010) 284–7.

67
*Aydin v Turkey* App no 23178/94 (25 September 1997) para 86; *Maslova and Nalbandov v Russia* App no 839/02 (24 January 2008) para 107.

68 See eg B Rainey, E Wicks and C Ovey, *Jacobs, White, and Ovey: the European Convention on Human Rights* (7th edn, OUP 2017) 187–91. However, see *Cestaro v Italy* App no 6884/11 (7 April 2015) paras [171]–[176].

69
*Ireland v UK* (n 54) para 167.

70
*Lyalyakin v Russia* App no 31305/09 (12 March 2015).

71
*Aghdgomelashvili and Japaridze v Georgia* App no 7224/11 (8 October 2020) paras 48–9; *Wiktorko v Poland* App no 14612/02 (31 March 2009) paras 54–5; *Iwańczuk v Poland* App no 25196/94 (15 November 2001) paras 54–60; *Valašinas v Lithuania* App no 44558/98 (24 July 2001) para 117.

72 The important role of qualitative considerations has emerged more clearly in recent case law under art 3. As the ECtHR held in para 42 of *Aghdgomelashvili* (n 70), treatment ‘can be qualified as “degrading” … if it shows a lack of respect for, or diminishes, human dignity’.

73 Waldron (n 65) 308–9.

74 Ibid.

75 G Vlastos, ‘Justice and Equality’ in J Waldron (ed), *Theories of Rights* (OUP 1984) 41–77.

76 J Waldron, *Dignity, Rank, and Rights* (OUP 2012).

77 C Taylor, ‘The Politics of Recognition’ in A Gutman (ed), *Multiculturalism and the Politics of Recognition* (Princeton UP 1994) 26–7.

78 Vlastos (n 74) 54.

79 It is neither possible nor necessary to offer a full account of human dignity for the purposes of this claim, namely that treatment that disrespects the equal moral status of all individuals violates dignity. See N Mavronicola, ‘Torture and Othering’ in BJ Goold and L Lazarus (eds), *Security and Human Rights* (Hart Publishing 2019) 27–52; E Webster, *Dignity, Degrading Treatment and Torture in Human Rights Law* (Routledge 2018) ch 2.

80
*Bouyid v Belgium* App no 23380/09 (Grand Chamber, 28 September 2015) para 112.

81 ibid para 107. The vulnerability of an applicant is an aggravating factor when assessing whether ill-treatment is severe enough to fall under art 3. See L Peroni and A Timmer, ‘Vulnerable Groups: The Promise of an Emerging Concept in European Human Rights Convention Law’ (2013) 11 ICON 1056.

82 C Heri, ‘Shaping Coercive Obligations through Vulnerability: The Example of the ECtHR’ in L Lavrysen and N Mavronicola (eds), *Coercive Human Rights: Positive Duties to Mobilise the Criminal Law under the ECHR* (Hart Publishing 2020) 93–116; A Timmer, ‘A Quiet Revolution: Vulnerability in the European Court of Human Rights’ in MA Fineman and A Grear (eds), *Vulnerability: Reflections on a New Ethical Foundation for Law and Politics* (Ashgate 2013) 162–4.

83
*Bouyid* (n 79) para 107.

84 ibid paras 88 and 100.

85 ibid para 103.

86 ibid para 104.

87 ibid para 106.

88 In *Bouyid*, the ECtHR emphasises the ‘strong link’ between human dignity and degrading treatment, and that even in the absence of ‘actual bodily injury or intense physical or mental suffering’ treatment showing ‘a lack of respect for or diminishing human dignity’ may be classed as degrading. See *Bouyid* (n 79) paras 87 and 90. See also Webster (n 78) 105–17; N Mavronicola, ‘Bouyid and Dignity’s Role in Article 3 ECHR’ (*Strasbourg Observers*, 8 October 2015) <https://strasbourgobservers.com/2015/10/08/bouyid-and-dignitys-role-in-article-3-echr/> accessed 26 May 2021.

89 Hellman (n 16) 34–58; J Hampton, ‘Forgiveness, Resentment and Hatred’ in JG Murphy and J Hampton (eds), *Forgiveness and Mercy* (CUP 1988) 52.

90 The ECtHR suggests so in *Bouyid* (n 79) para 87.

91
*Identoba and Others v Georgia* App no 73235/12 (12 May 2015) para 71.

92 The police authorities had been informed ‘well in advance’ of the LGBT community’s intention to hold a march in the centre of Tbilisi on 17 March 2012. See ibid para 72.

93 ibid paras 69 and 70.

94 ibid para 65. See also *Eremia v the Republic of Moldova* App no 3564/11 (28 May 2013) para 54; *Gäfgen v* *Germany* App no 22978/05 (Grand Chamber, 1 June 2010) para 103.

95
*Identoba* (n 90) paras 68 and 70.

96 ibid.

97
*Identoba* (n 90) para 70 (emphasis added).

98 ibid para 67. See also *Begheluri and Others* App no 28490/02 (7 October 2014) para 173.

99
*MC* *and* *AC* *v Romania* App no 12060/12 (12 June 2016) paras 116–18.

100
*Aghdgomelashvili* (n 70) paras 44 and 48–9.

101
*Identoba* (n 90) para 65. The ECtHR has repeatedly held that racial discrimination is a ‘special affront to human dignity’ and can as such amount to degrading treatment under art 3. See *Moldovan v Romania* App no 41138/98 (12 July 2005) para 110; *Nachova and Others v Bulgaria* App nos 43577/98 and 43579/98 (Grand Chamber, 6 July 2005), para 145.

102 UN Committee Against Torture, ‘General Comment No 2’ (CAT/C/GC/2, 24 January 2008) paras 20–1. Discrimination also features in the definition of torture in art 1 CAT.

103
*East African Asians v United Kingdom* (1981) 3 EHRR 76.

104 ibid para 196.

105 ibid para 207.

106 ibid para 208.

107
*Cyprus v Turkey* App no 25781/94 (10 May 2001) paras 306–9.

108 ibid paras 245 and 292–3.

109
*Dudgeon v United Kingdom* App no 7525/76 (Grand Chamber, 22 October 1981) para 52.

110
*X v Turkey* App no 24626/09 (27 May 2013).

111
*Vallianatos and Others v Greece* App nos 29381/09 and 32684/09 (Grand Chamber, 7 November 2013) para 77.

112
*Smith and Grady v United Kingdom* App nos 33985/96 and 33986/96 (27 September 1999) para 121.

113
*X* (n 109).

114 ibid para 56.

115 ibid para 53.

116 ibid para 57.

117 P Johnson and S Falcetta, ‘Sexual Orientation Discrimination and Article 3 of the European Convention on Human Rights: Developing the Protection of Sexual Minorities’ (2018) 43 ELR 167, 175–6.

118
*Identoba* (n 90) para 70; *MC* (n 98) paras 117–19.

119 UN Human Rights Council, ‘Annual Report of the United Nations High Commissioner for Human Rights: Discriminatory Laws and Practices and Acts of Violence Against Individuals Based on their Sexual Orientation and Gender Identity’ (19th Session, A/HRC/19/41, 17 November 2011) para 34.

120 Of course, being an aggravating factor, the ‘public nature’ factor is neither necessary nor sufficient to find a case of discrimination in violation of art 3. See eg *Lyalyakin* (n 69) para 69; *Svinarenko and Slyadnev v Russia* App nos 32541/08 and 43441/08 (Grand Chamber, 17 July 2014) para 115.

121 The approach of the ECtHR is similar to the approach of the UN CAT in this regard. See UN General Assembly, ‘Extra-Custodial Use of Force and the Prohibition of Torture and Other Cruel, Inhuman or Degrading Treatment’ (A/72/178, 20 July 2017).

122
*Tyrer v United Kingdom* App no 5856/72 (25 April 1978) para 33.

123 In cases involving the rights of transgender people, the ECtHR has held that respect for dignity requires the protection of moral security. See *Van Kück v Germany* App no 35869/97 (12 June 2003) para 69; *I* *v United Kingdom* App no 25680/94 (11 July 2002) para 70; *Christine Goodwin v United Kingdom* App no 28957/95 (11 June 2002) para 90.

124 J Wolfendale, ‘Moral Security’ (2017) 25 Journal of Political Philosophy 238, 244; Wolff (n 34). See also C Nikolaidis, ‘Unravelling the Knot of Equality and Privacy in the European Court of Human Rights and the US Supreme Court: From *Isonomia* to *Isotimia*’ [2018] HRLR 719, 736.

125 GC Lury, *The Anatomy of Racial Inequality* (Harvard UP 2002) 58.

126 T Khaitan, *A Theory of Discrimination Law* (OUP 2015) 126–8; S Bagenstos, ‘“Rational Discrimination”, Accommodation, and the Politics of (Disability) Civil Rights’ (2003) 89 Va L Rev 825.

127 S Moreau, ‘What Is Discrimination’ (2010) 38 Philosophy & Public Affairs 143, 177–8.

128 On the contrary, degrading discrimination can take place in a prison, see eg *X* (n 109).

129 ibid paras 72–3.

130
*Svinarenko* (n 119) para 114; *V v United Kingdom* App no 24888/94 (Grand Chamber, 16 December 1999) para 71.

131
*Gäfgen* (n 93) para 107.

132 ibid

133
*Ireland* (n 54) para 167.

134
*Tysiąc v Poland* App no 5410/03 (20 March 2007) para 67.

135
*Lyalyakin* (n 69) para 69.

136
*Chember v Russia* App no 7188/03 (3 July 2008) para 49.

137 In the jurisprudence of the ECtHR, degradation is closer to being demeaned, in the sense that the person wronged does not have to feel that their moral status has been lowered. See Hampton (n 88) 44–5.

138
*Poltoratskiy v Ukraine* App no 38812/97 (29 April 2003) para 131.

139 As the ECtHR held in *MSS*, it ‘may well suffice that the victim is humiliated in his own eyes, even if not in the eyes of others’: *MSS* *v Belgium and Greece* App no 30696/09 (Grand Chamber, 21 January 2011) para 220.

140 ‘Report of the Special Rapporteur on the Question of Torture’ (n 15) para 24.

141
*Maslova and Nalbandov v Russia* App no 839/02 (24 January 2008); see also *Aydin* (n 66) para 86. Threats of violence can constitute torture; see *Selmouni v France* App no 25803/94 (28 July 1999) para 101. Torture covers both physical pain and mental suffering; see *Gäfgen* (n 93) para 108.

142
*Jalloh v Germany* (n 55) para 67.

143
*Vladimir Romanov v Russia* App no 41461/02 (24 July 2008) paras 67–70.

144
*Cestaro* (n 67) paras 182 and 189. See also *Dedovski and Others v Russia* App no 7178/03 (15 May 2008) paras 82–3.

145 ‘Forcible’ is used to mark cases where a person undergoing ‘conversion therapy’ is not free to leave or stop the ‘therapy’. ‘Forcible’ covers those who knew that they would not be able to leave it if they changed their mind, even if they consented to the ‘conversion therapy’.

146
*Herczegfalvy v Austria* App no 10533/83 (24 September 1992) para 82.

147
*R (on the application of Wilkinson) v The Responsible Medical Officer Broadmoor Hospital* [2001] EWCA Civ 1545, [2001] 1 WLR 419, paras 77–80 (Hale LJ). This applies to both capacitated and incapacitated patients.

148 ibid para 79; *Herczegfalvy* (n 145) para 82. There is a duty to give reasons whenever a patient should undergo medical treatment without their consent. See *R (Wooder) v Feggetter* [2002] EWCA Civ 554, [2002] 3 WLR 591; *R (B) v SS (Responsible Medical Officer)* [2006] EWCA Civ 28, [2006] 1 WLR 810, para 50.

149
*Herczegfalvy* (n 145) para 82.

150
*Wilkinson* (n 146) para 55.

151 ‘Report of the Special Rapporteur’ (n 10) para 23; see also Bartlett, Smith and King (n 8).

152 The compulsion of the medical treatment overshadows whether the patient had the capacity to consent to the treatment. See *Wilkinson* (n 146) para 79; *R (B) v SS (Responsible Medical Officer)* [2006] EWCA Civ 28, [2006] 1 WLR 810, para 50.

153 This does not include counselling which seeks to provide acceptance, support, facilitation and understanding of a person’s sexual and gender identity. See n 17 above.

154 So, legal intervention against ‘conversion therapy’ is justified, at least in part, by appeal to the states of affairs it promotes. This (broadly) consequentialist view is different to rule utilitarianism because it is unconcerned with benefit maximisation. See TM Scanlon, ‘Rights, Goals and Fairness’ in TM Scanlon (ed), *The Difficulty of Tolerance* (CUP 2003) 33–9.

155 The prohibition of discrimination is partly a response to the subordinating meaning of ‘institutionalised humiliation’ conveyed by certain forms of disadvantageous treatment. See JM Balkin and RB Siegel, ‘The American Civil Rights Tradition: Anticlassification or Antisubordination?’ (2004) 58(9) U Miami L Rev 9.

156 Expressive harms can directly injure, and function differently from ideological or purely subjective injuries. ‘Expressive Harms and Standing’ (1999) 112 Harv L Rev 1313; RH Pildes and RG Niemi, ‘Expressive Harms, “Bizarre Districts”, and Voting Rights: Evaluating Election-District Appearances After *Shaw v Reno*’ (1993) 92 Mich L Rev 483.

157 Subordinated groups do not choose the social meanings imposed on them by society’s institutions, such as religious groups or medical experts. L Melling, ‘Religious Refusals to Public Accommodations Laws: Four Reasons to Say No’ (2015) 38 Harvard Journal of Law and Gender 177; M Lim and L Melling, ‘Inconvenience or Indignity? Religious Exemptions to Public Accommodations Laws’ (2014) 22 J L & Pol’y 705.

158 See eg N Mavronicola, *Torture, Inhumanity and Degradation under Article 3 of the ECHR: Absolute Rights and Absolute Wrongs* (Hart Publishing 2021) ch 3; M Nowak and E McArthur, ‘The Distinction Between Torture and Cruel, Inhuman or Degrading Treatment’ (2006) 16 Torture 147.

159 The Yogyakarta Principles Plus 10, Principle 10 E. Although the Yogyakarta Principles are not legally binding, they are highly influential as they remain the most comprehensive identification of state human rights obligations in relation to sexual orientation and gender identity. See M O’Flaherty and J Fisher, ‘Sexual Orientation, Gender Identity and International Human Rights Law: Contextualising the Yogyakarta Principles’ (2008) 8 HRLR 207, 237–47.

160 UN Committee Against Torture, ‘Concluding Observations on the Seventh Periodic Report of Ecuador’ (CAT/C/ECU/CO/7, 11 January 2017) para 49.

161 UN Committee Against Torture, ‘Concluding Observations on the Fifth Periodic Report of China’ (CAT/C/CHN/CO/5, 3 February 2016) para 55.

162 ibid.

163 ibid para 56.

164 ibid. See also UN HRC (n 118) para 56.

165 Apart from ‘framework’ and operational positive duties, art 3 also gives rise to investigative duties. Those fall outside the scope of this article.

166 See eg *Đorđević v Croatia* App no 41526/10 (24 July 2012) para 138; *Beganović v Croatia* App no 46423/06 (25 June 2009) para 71; *Nachova* (n 100) para 96; *A v United Kingdom* (1998) 27 EHRR 611, para 22.

167
*Šečić v Croatia* App no 40116/02 (31 May 2007) para 53; *Moldovan and Others v Romania* App nos 41138/98 and 64320/01 (12 July 2005) para 98; *MC* *v Bulgaria* App no 39272/98 (4 December 2003) para 151. See also *Gezer v Secretary of State for the Home Department* [2004] EWCA Civ 1730, [2005] HRLR 7. See also, *mutatis mutandis*, *Commissioner of Police of the Metropolis v DSD* [2018] UKSC 11, [2019] AC 196, para 88 (Lord Neuberger).

168
*Osman v United Kingdom* App no 23452/94 (Grand Chamber, 28 October 1998) para 116. On the application of the *Osman* test in the context of art 3, see *Đorđević* (n 165). See also *Z and Others v United Kingdom* App no 29392/95 (Grand Chamber, 10 May 2001) para 255. The UK courts apply the *Osman* test in cases involving complaints under art 3. See *DSD* (n 166) paras 92–8 (Lord Neuberger); *R (Munjaz) v Ashworth Hospital Authority* [2005] UKHL 58, [2006] 2 AC 148, paras 78–80.

169
*Đorđević* (n 165) para 139.

170 Mavronicola (n 58) 732–5.

171
*Osman* (n 167) para 116.

172 UN Committee on the Elimination of Discrimination Against Women, ‘VK v Bulgaria’ (Communication no 20/2008, 15 October 2008) para 9.8.

173
*Volodina v Russia* App no 41261/17 (9 July 2019) para 86.

174 ibid.

175 Separate opinion of Judge Pinto de Albuquerque in *Volodina* (n 172) para 12.

176 This is congenial to the presumption set in *Re E*, namely that the authorities knew or ought to have known about the existence of a real risk of ill-treatment whenever a breach has occurred, and then recurred, over a period of time. See *Re E v Chief Constable of the Royal Ulster Constabulary and another* [2008] UKHL 66, [2009] 1 AC 536.

177 For a critical appraisal of this presumption, see L Lazarus, ‘Positive Obligations and Criminal Justice: Duties to Protect or Coerce’ in L Zadner and J Roberts (eds), *Principles and Values in Criminal Law and Criminal Justice* (OUP 2012) 135–57; F Tulkens, ‘The Paradoxical Relationship between Criminal Law and Human Rights’ (2011) 9 JICJ 577.

178 N Mavronicola, ‘Coercive Overreach, Dilution and Diversion: Potential Dangers of Aligning Human Rights Protection with Criminal Law (Enforcement)’ in Lavrysen and Mavronicola (n 81) 183–202.

179
*MC* *v Bulgaria* (n 166) para 166; *X and Y v The Netherlands* App no 8978/80 (26 March 1985).

180
*M and C v Romania* App no 29032/04 (27 September 2011).

181
*Cestaro* (n 67) para 225.

182
*Myumyun v Bulgaria* App no 67258/13 (3 November 2015) para 77.

183
*Volodina* (n 172) para 81.

184 L Lavrysen, ‘Positive Obligations and the Criminal Law: A Bird’s-Eye View on the Case Law of the European Court of Human Rights’ in Lavrysen and Mavronicola (n 81) 29–55, 43.

185
*Myumyun* (n 181) para 74.

186
*Volodina* (n 172) para 81.

187 That is enough for a treatment to qualify as ‘degrading’ under art 3 ECHR. See *Identoba* (n 90) para 65.

188 See nn 183 and 184.

189 Malta, for instance, has criminalised the provision of ‘conversion therapy’ (see n 2).

190
*Mitkus v Latvia* App no 7259/03 (2 October 2010) para 76. That might not be the case though for grossly negligent violations of art 3. See K Kamber, *Prosecuting Human Rights Offences: Rethinking the Sword Function of Human Rights Law* (Brill 2017) ch 1.

191 Specifically in the UK, the authorities cannot claim that they were unaware of the risks of ‘conversion therapy’. The 2018 National LGBT Survey showed that significant numbers of LGBTIQ+ people have been offered ‘conversion therapy’ (see n 9 above) whereas in 2017 the NHS and leading medical professional bodies signed a Memorandum of Understanding on ending ‘conversion therapy’; see BACP and others (n 4) para 3.

192 See nn 1 and 9 above.

193 As is the case, for instance, in Albania (n 3).

194 See n 17 above.

195 On the positive obligation under art 3 regarding children, see *O’Keeffe v Ireland* App no 35810/09 (Grand Chamber, 28 January 2014) paras 144–52.

196 See eg *Code of Practice for Victims of Crime* (Ministry of Justice 2015).

